# Resolving the smell of wood - identification of odour-active compounds in Scots pine (*Pinus sylvestris* L.)

**DOI:** 10.1038/s41598-018-26626-8

**Published:** 2018-05-29

**Authors:** Linda Schreiner, Patrick Bauer, Andrea Buettner

**Affiliations:** 1 Friedrich-Alexander-Universität Erlangen-Nürnberg, Chair of Aroma and Smell Research, Emil Fischer Center, Department of Chemistry and Pharmacy, Henkestrasse 9, 91054 Erlangen, Germany; 2 Department Sensory Analytics, Fraunhofer Institute for Process Engineering and Packaging IVV, Giggenhauser Straße 35, 85354 Freising, Germany

## Abstract

Being one of the most common trees in forests, *Pinus sylvestris* L. is a frequently used raw material for wood products. Its specific odour is, however, mostly unresolved to date. Accordingly, we investigated Scots pine wood samples grown in Germany for their main odorant composition. We employed dedicated odorant analysis techniques such as gas chromatography-olfactometry (GC-O) and aroma extract dilution analysis (AEDA) and successfully detected 44 odour-active compounds; of these, 39 substances were successfully identified by gas chromatography-mass spectrometry/olfactometry (GC-MS/O) and two-dimensional gas chromatography-mass spectrometry/olfactometry (2D-GC-MS/O). Among the main odorants found were (*E,E*)-nona-2,4-dienal, vanillin, phenylacetic acid, 3-phenylpropanoic acid, δ-octalactone and α-pinene, all of them having been detected with high flavour dilution factors during GC-O analyses. The majority of the identified odorants were fatty acid degradation products, plus some terpenoic substances and odorous substances resulting from the degradation of lignin. Although some of the detected substances have previously been reported as constituents of wood, 11 substances are reported here for the first time as odour-active compounds in wood, amongst them heptanoic acid, γ-octalactone, δ-nonalactone and (*E,Z,Z*)-trideca-2,4,7-trienal.

## Introduction

With over 100 different species widely distributed all over the world, the pine tree (*Pinus*) represents the largest genus of the conifers^1^. The undemanding requirements with regard to temperature, light conditions and soil quality allow the pine to grow in many parts of the world. Accordingly, the geographical distribution of the pine tree ranges from North America, Europe and Asia to a few south-east Asian countries, and even extends to some tropical regions. The fast and straight growth of their trunks makes the pine an ideal tree for wood production and its subsequent processing. The wood’s specific properties with respect to elasticity and strength as well as its decorative appearance predetermine its usage as a building material or for the fabrication of furniture and commodities. In German forests *Pinus sylvestris* L. is one of the most common trees and a popular material for the manufacture of wood products^2^.

Due to its natural appearance and specific odour, pine wood products such as toys are often preferred by consumers to those made from synthetic materials; the pine tree smell is commonly referred to as being natural, pleasant and harmonizing^3^. Moreover, wood odour is often associated with a positive impact on health. Essential oil from various pinaceaous trees has been demonstrated to exert relaxing effects: the inhalation of the essential oil of *Abies sibirica*, for example, reduced the level of arousal in people after performing a video display terminal work task, and helped the participants recover from mental fatigue^4^. The authors concluded that the essential oil may prevent mental health problems such as sleep disorders^4^. Such observations are further supported by animal studies where the essential oil of *Abies sachalinensis* has been shown to have an anxiolytic effect on mice^5^. Furthermore, investigations in rats revealed the ability of plant-derived odours, such as α-pinene, to calm stress-induced hyperthermia^6^. Additionally, it was shown that taking in the atmosphere of forest (Shinrin-yoku) impacts cerebral activity and salivary cortisol measurements. Based on these observations, the authors stated that these physiological effects are related to relaxation of the human body and spirit^7^.

Despite such reports on potential positive physiological effects of wood odour on humans, there is still a dearth of information about the underlying odorous constituents. Previous studies mainly focused on the general identification of volatile organic compounds (VOCs) in wood, wood products or the respective essential oils^8–12^. For example, Weissbecker *et al*.^12^ reported volatile substances in the headspace of pine wood (*Pinus sylvestris* L.) with the aim of detecting substances that are recognized by insects. Thus, stimuli were identified by means of gas chromatography-mass spectrometry coupled with electroantennographic detection (GC-MS/EAD), meaning that the insect antennas were utilized for the targeted detection of the compounds. The authors showed that insect antennae detection mainly yielded terpenoids, aliphatic aldehydes and alcohols, especially hexanal and α-pinene. Similar results were obtained by Uçar *et al*.^9^ using an untargeted approach, employing gas chromatography-mass spectrometry (GC-MS) in their study of volatiles from *Pinus nigra* Arnold. They identified general volatile compounds in wood, but none of these were specifically tested with regard to their odour activity and contribution to the overall wood odour profile.

Studies on wood odorants are few in number, and have primarily focused on the identification of odorants in wood of potential relevance for the production of alcoholic beverages^13,14^. These studies predominantly focused on toasted woods, whereas natural wood odour has been largely overlooked in scientific studies. To remedy this, we recently identified the main odorants in incense cedar wood which is commonly used for everyday products such as pencils^15^. The potent contributors to this type of wood smell were previously unknown and were identified in our study using sophisticated odorant analysis techniques. To extend our research on wood odorants, the current study aimed to elucidate the smell of pine wood by analysing the odour-active substances in three *Pinus sylvestris* L. samples. As in our previous study, a targeted approach was adopted, combining state-of-the-art odorant analysis methods with human-sensory evaluation of the odorous compounds. The most potent odorants were rated via aroma extract dilution analysis (AEDA) according to Grosch^16^ and identified by one- and two-dimensional high resolution gas chromatography-olfactometry/mass spectrometry. The findings are an important step towards a better understanding of compounds that generate typical wood smells.

## Results

### Odour profiles and sensory evaluation

**Figure 1:**
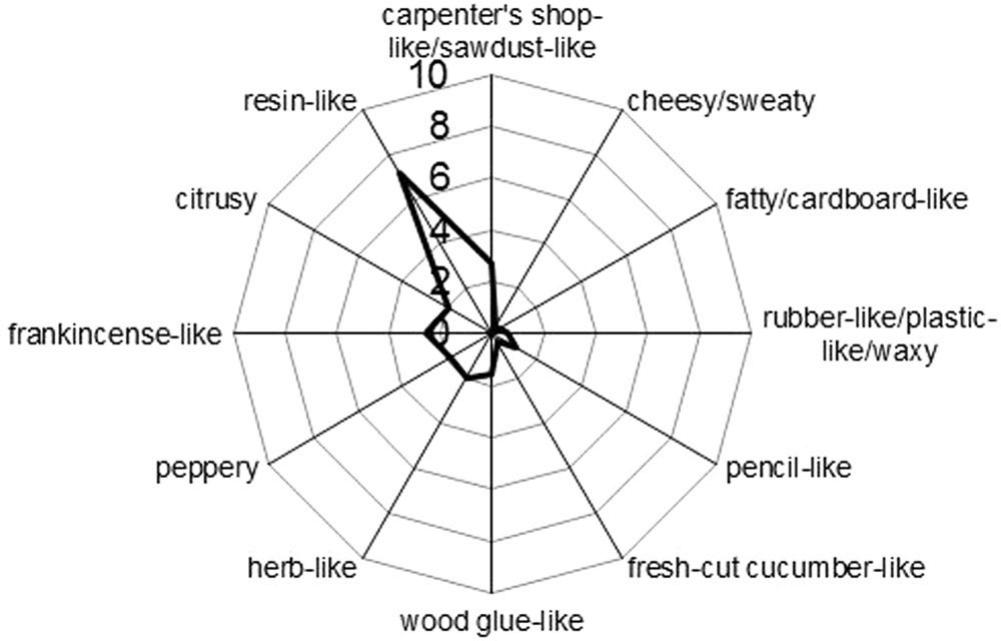
Odour profile of pine wood sample 1.

In a first step, 30 untrained panellists were asked to rate the pleasantness and intensity of the pine wood. Since these sensory tests were performed to give a preliminary overview on the pine wood odour, only wood splints of one of the *Pinus sylvestris* L. samples were presented to the panellists. On a 10 cm visual analogue scale from 0 (no perception) to 10 (very intense) the pine wood was scored with a high intensity of 7.8 ± 1.6 and a slightly positive hedonic rating of 6.2 ± 2.7 on a scale from 0 (very unpleasant) to 10 (very pleasant). In order to acquire a detailed odour profile (see Fig. 1) of the sample, a panel of extensively trained and tested persons was asked to select odour attributes in consensus for the description of the odour impressions of the samples. The attributes chosen were *carpenter’s shop-like/sawdust-like, pencil-like, fatty/cardboard-like, herb-like, fresh-cut cucumber-like, cheesy/sweaty, rubber-like/plastic-like/waxy, wood glue-like, peppery, frankincense-like, citrusy* and *resin-like*. In a second evaluation, the intensity of each attribute was rated on a 10 cm visual analogue scale from 0 (no perception) to 10 (strong perception). The odour of the *Pinus sylvestris* L. sample was described as mainly *resin-like* with a high intensity of 7.2. The attributes *frankincense-like* (2.6) and *carpenter’s shop-like/sawdust-like* (2.7) were rated with medium intensities. The attributes *wood glue-like, herb-like, peppery* and *citrusy* were recorded with intensities between 1.5 and 2.0. The remaining attributes reached intensities of 0.3 to 1.0 only, and were, accordingly, of minor relevance. To elucidate the underlying odorants representing these impressions in pine wood odour, the odour-active substances were characterised as described in the next section.

### Characterisation of the odorant composition

To detect possible differences in odorants between particular trees, two additional *Pinus sylvestris* L. samples were analysed by GC-O. This led to the detection of a number of odour-active compounds in the undiluted extracts, with detection of a total of 44 odour-active compounds in the course of AEDA spanning the flavour dilution (FD) factor range from 9 to 729. Of these, 39 substances could be aligned with reference compounds based on the criteria given in Table 1. Where no reference was available, this is indicated in the information provided. All odorants could be olfactorily detected in at least two of the three wood samples under investigation, albeit with somewhat varying FD factors for individual odorants within the different samples, thereby not exceeding differences of more than three dilution steps.

**Table 1:** Odour-active compounds, their retention indices and FD factors on a DB-FFAP capillary, and their odour qualities as identified in three *Pinus sylvestris* L. samples.

No	Substance	RI DB-FFAP	Odour quality	FD DB-FFAP			Identification^a^
				Sample 1	Sample 2	Sample 3	
1	Pentanal	1000	green, grassy	9	27	1	RI, O, RC
**2**	**α-Pinene**	**1032**	**woody, resinous**	**729**	**729**	**243**	**MS, RI, O, RC**
3	Hexanal	1088	green, grassy	243	243	27	MS, RI, O, RC
4	Octanal	1288	citrusy	9	243	27	MS, RI, O, RC
5	1-Octene-3-one	1300	mushroom-like	9	27	27	MS, RI, O, RC
6	Nonanal	1392	citrusy	9	27	9	MS, RI, O, RC
7	(*E*)-Oct-2-enal	1431	fatty	27	27	27	MS, RI, O, RC
8	Acetic acid	1450	vinegar-like	9	81	27	RI, O, RC
9	(*Z*)-Non-2-enal	1493	fatty	81	n.d.	27	RI,O
10	(*E*)-Non-2-enal	1526	fatty	243	243	243	MS, RI, O, RC
11	Linalool	1543	citrusy	243	27	n.d.	MS, RI, O, RC
12	Fenchol	1580	mouldy	9	n.d.	9	MS, RI, O, RC
13	*(E,Z)*-Nona-2,6-dienal	1586	cucumber-like	n.d.	27	27	RI, O, RC
14	*(E)*-Dec-2-enal	1610	fatty	243	243	n.d.	RI, O, RC
15	Butanoic acid	1629	cheesy	81	27	27	MS, RI, O, RC
16	2-Butyl-2-octenal	1643	sweetish, metallic, citrus	n.d.	9	9	MS
17	3-Methylbutanoic acid	1677	cheesy	9	3	9	RI, O, RC
**18**	***(E,E)*** **-Nona-2,4-dienal**	**1700**	**fatty**	**729**	**729**	**243**	**MS, RI, O, RC**
19	Borneol	1705	mouldy	9	n.d.	27	MS, RI, O, RC
20	unknown	1718	minty, fresh	27	81	9	
**21**	**Pentanoic acid**	**1736**	**cheesy**	**27**	**729**	**27**	**MS, RI, O, RC**
**22**	***(E,E)*** **-Deca-2,4-dienal**	**1810**	**fatty**	**729**	**n.d**.	**81**	**MS, RI, O, RC**
23	Hexanoic acid	1848	spit-like, animal-like, plastic-like	27	81	81	MS, RI, O, RC
24	5-Butyloxolan-2-one *(γ*-Octalactone)	1917	sweetish, coconut-like	n.d.	81	27	MS, RI, O, RC
**25**	**Heptanoic acid**	**1942**	**pepperoni-like, plastic-like**	**729**	**81**	**81**	**MS, RI, O, RC**
**26**	**6-Propyloxan-2-one (** ***δ*** **-Octalactone)**	**1984**	**coconut-like**	**729**	**243**	**243**	**MS, RI, O, RC**
27	5-Pentyloxolan-2-one *(γ*-Nonalactone)	2037	peach-like	81	243	81	MS, RI, O, RC
**28**	***(E,Z,Z)*** **-Trideca-2,4,7-trienal**	**2058**	**fruity, blood-like, metallic**	**9**	**729**	**27**	**RI, O, RC**
29	4-Methylphenol (p-Cresol)	2089	horse-like	27	3	27	MS, RI, O, RC
30	6-Butyloxan-2-one *(δ*-Nonalactone)	2089	sweet, coconut-like	243	81	n.d.	RI, O, RC
31	5-Hexyloxolan-2-one *(γ*-Decalactone)	2156	peach-like	9	1	9	RI, O, RC
32	Nonanoic acid	2164	leather-like, artificial, soapy	n.d.	243	27	RI, O, RC
33	Sotolone	2212	savoury	243	27	81	RI, O, RC
**34**	**α-Bisabolol**	**2255**	**balsamic, pepper-like**	**9**	**729**	**243**	**MS, RI, O, RC**
35	unknown	2300	leather-like	81	27	n.d.	
36	5-Octyloxolan-2-one (γ-Dodecalactone)	2388	peach-like	9	9	9	RI, O, RC
37	Dodecanoic acid	2489	perfume-like, soapy	9	9	27	RI, O, RC
38	unknown	2538	rubber-like	n.d.	27	243	
**39**	**Phenylacetic acid**	**2567**	**honey-like**	**729**	**729**	**729**	**RI, O, RC**
**40**	**Vanillin**	**2594**	**vanilla-like**	**729**	**729**	**729**	**MS, RI, O, RC**
**41**	**3-Phenylpropanoic acid**	**2640**	**vomit-like, fruity**	**729**	**243**	**243**	**RI, O, RC**
42	unknown	2878	androstenone-like, perfume-like	81	81	9	
43	unknown	2927	androstenone-like, perfume-like	27	243	243	
**44**	**Thymoquinone**	**3100**	**pencil-like**	**729**	**n.d**.	**81**	**MS, RI, O, RC**

The most potent odorants are indicated in bold print.

^a^Identification methods: MS: mass spectrum, RI: retention indices, O: odour quality, RC: comparison of all data with reference compounds; n.d. not detected by means of olfactory detection in the most concentrated sample referring to FD 1.

Twelve substances were detected with FD factors of 729 in at least one of the investigated samples. These most potent substances were: the woody smelling 2,6,6-trimethylbicyclo[3.1.1]hept-2-ene (α-pinene), the fatty smelling (*E,E)*-nona-2,4-dienal and (*E,E)*-deca-2,4-dienal, the cheesy smelling pentanoic acid, the plastic-like smelling heptanoic acid, and the coconut-like smelling 6-pentyloxan-2-one (δ-octalactone). Furthermore, 6-methyl-2-(4-methylcyclohex-3-en-1-yl)hept-5-en-2-ol (α-bisabolol; balsamic, pepper-like), the vanilla-like smelling 4-hydroxy-3-methoxybenzaldehyd (vanillin), the vomit-like smelling 3-phenylpropanoic acid and the pencil-like smelling 2-isopropyl-5-methylbenzo-1,4-quinone (thymoquinone) were detected with the highest FD factors and could be successfully identified via alignment with their respective reference compounds, based on their mass spectrometric data as well as their chromatographic and olfactometric characteristics (see Table 1). (*E,Z,Z*)-Trideca-2,4,7-trienal (blood-like smell) and phenylacetic acid with a honey-like impression were also detected with highest FD factors and were identified by comparing their odour qualities and retention indices with respective reference compounds. Of these most potent odorants, the following are reported here for the first time as odorous constituents in wood of *Pinus* species: (*E,E)*-nona-2,4-dienal, (*E,E)*-deca-2,4-dienal, pentanoic acid, heptanoic acid, δ-octalactone, α-bisabolol, vanillin, 3-phenylpropanoic acid, thymoquinone, (*E,Z,Z)*-trideca-2,4,7-trienal and phenylacetic acid.

Using GC-MS/O and 2D-GC-MS/O, mass spectrometric confirmation was achieved for 23 substances. In addition to the aforementioned substances representing the highest FD factors, the following odour-active substances in *Pinus sylvestris* L. were identified by comparing their mass spectra, retention indices and odour qualities with respective reference compounds: hexanal, octanal, 1-octene-3-one, nonanal, (*E*)-oct-2-enal, linalool, fenchol, butanoic acid, borneol, hexanoic acid, γ-octalactone, γ-nonalactone and p-cresol.

For 2-butyl-2-octenal, mass spectrometric alignment was based on comparison with data in the NIST library since the respective original reference compound was not available. For the remaining substances, tentative identification was based on their retention indices on two analytical capillaries having different polarities, their odour qualities, and comparison of these data with original reference compounds analysed in parallel. Mass spectrometric detection was not achieved for these substances since their concentrations in the sample were too low and additionally the co-elution of odourless substances posed an obstacle for unambiguous identification; however, olfactometric detection of these compounds, at times with quite pronounced FD factors (FD above 9) demonstrated their high odour potency.

Five compounds smelling minty, leather-like, androstenone-like/perfume-like and rubber-like could not be identified. The minty and leather-like substances reached a maximum FD factor of 81, whereas the other substances were detected with a maximum FD of 243.

## Discussion

In total 39 odorants of the 44 detected compounds were identified using GC analyses. The number of odorants found in *Pinus sylvestris* L. can be arbitrarily increased by injecting higher concentrations of the aroma extract but experience has shown that in most cases the 20 most potent odorants of an aroma extract are sufficient to reconstitute the overall odour impression of the sample^17^.

Most of the substances found in the *Pinus sylvestris* L. samples were present in all three samples. Fourteen substances occurred in two samples only, whereas each of the odour-active substances was present in at least two samples. Generally, non-detection by the sniffing experiments does not rule out the presence of the respective odorants in the wood sample, it means that it is present in concentrations below the perceptual level with regards to FD 1 (the most concentrated sample). Moreover, there were mostly no large variations in FD factors between the samples. Experience shows that in natural samples FD-factors can vary within up to three FD stages^18,19^. Larger differences in FD factors may be due to age differences between the three trees or different external conditions such as light exposure and humidity.

The results of the sensory evaluation and the results obtained by gas chromatography showed close agreement: In the course of the sensory testing the highest intensity rating by the panellists was for the attribute *resin-like*. This odour impression is likely to be correlated with α-pinene, which smells *woody/ resinous*. It is interesting to note that α-pinene is one of the largest peaks in the chromatograms of each of the *Pinus sylvestris* L. samples. This, together with its relatively high odour potency, suggests that this terpene has a main impact on the overall odour impression of the wood samples. This finding is not surprising because α-pinene is long known to be an odorous constituent of pine wood^20^. Among the identified substances were many fatty smelling mono- and di-alkenals such as (*E)*- and (*Z)*-non-2-enal, (*E,E)*-nona-2,4-dienal and (*E,E)*-deca-2,4-dienal. These are reported here for the first time as odorants in *Pinus sylvestris* L. wood. Their smell impressions are likely to be represented by the *fatty/cardboard-like* note perceived during the sensory evaluation. The *citrusy* note can be assumed to result from octanal, linalool and nonanal, whereas the *green, grassy* smelling pentanal and hexanal as well as the *savoury-like* smelling sotolone are likely candidates for being responsible for the *herb-like* note. Furthermore, (*E,Z)*-nona-2,6-dienal (*cucumber-like*) and butanoic acid as well as 3-methylbutanoic acid (*cheesy*) can be associated with the *fresh-cut cucumber-like* and *cheesy/sweaty* odour impressions. The *pencil-like* smell can be traced back to thymoquinone and the *peppery* note to α-bisabolol. *Rubber-like/plastic-like/waxy* notes may stem from a *rubber-like* smelling unknown substance, and hexanoic acid or heptanoic acid (both smelling, inter alia, *plastic-like*). In contrast to these correlations between the sensory evaluations and GC-O results, some odour impressions from the sensory evaluation (*frankincense-like, carpenter’s shop-like/sawdust-like, wood glue-like*) could not be directly aligned with specific substances. These odour impressions may be a result of mixed odour effects and may be further influenced by additive, synergistic and suppressive effects of all odorous constituents present in the wood. Previous studies showed that unexpected odorous impressions can arise from mixtures of odorants that can be barely predicted, such as the grapefruit-like note induced by a mix of mainly citrusy and black current-like odour notes as described for grapefruit aroma^18,19^. Future studies involving quantification and reconstitution experiments would be required to answer this question.

**Figure 2:**
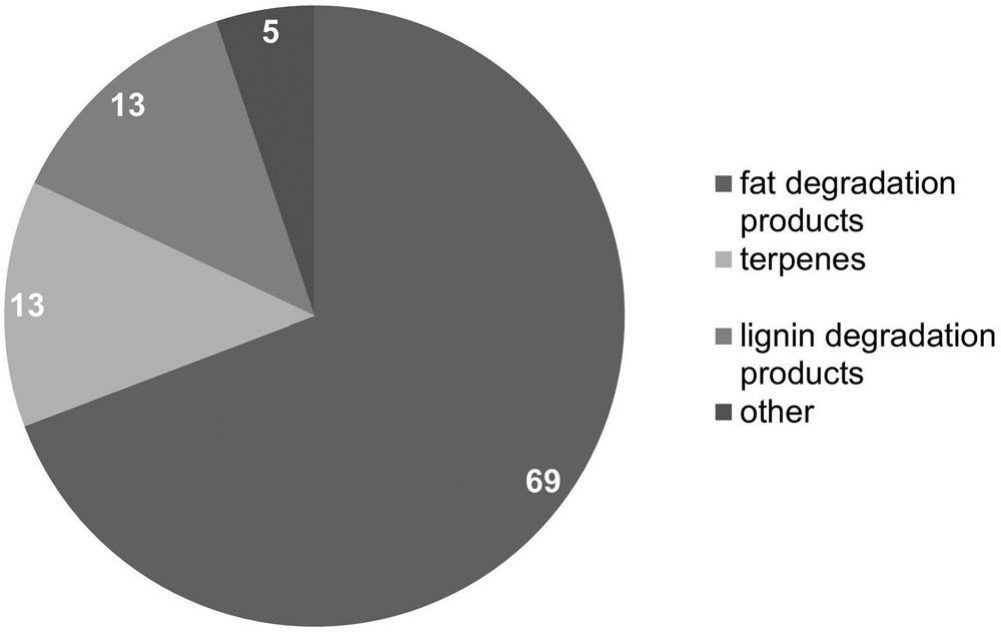
Percentage composition of the odour-active wood constituents [%] in *Pinus sylvestris* L. and potential source of origin as determined in the present study. Note: the percentages do not represent absolute quantitative data.

The identified odorants in the *Pinus sylvestris* L. samples belong to a variety of substance classes that exhibit great diversity of odour character. A large number of these odour-active substances stem from fatty acid degradation. Fatty acids and waxes are part of the extractable fraction in wood^21^. Since wood is naturally exposed to sun and air, degradation processes of fatty acids are promoted, resulting in various alkenals, ketones, alkylic acids and intramolecular esters. The odour impressions of these fatty acid degradation products range from *green, grassy* (pentanal, hexanal), *citrus-like* (octanal, nonanal, linalool) and *fatty* (e.g. (*E)*-non-2-enal, (*E,E)*-nona-2,4-dienal) to *cheesy* (butanoic acid) and *coconut-like/peach-like* (γ-octalactone, γ-nonalactone). Degradation products of fatty acids represent the largest group of odour-active constituents in wood with 69% (see Fig. 2).

Apart from the various fatty acid degradation products, a series of odorous molecules of terpenoic structure was found to be present in the *Pinus sylvestris* L. samples. Terpenes are part of the resin located in the resin channels in the heartwood and sapwood of conifers^22^. Representatives of this group are the monoterpenes α-pinene (*woody, resinous*), linalool (*citrusy*), borneol and fenchol (both *mouldy*) as well as the sesquiterpene α-bisabolol (*balsamic*, *peppery*).

Phenyl compounds are another prominent group of odour-active constituents in wood. They occur in wood due to the degradation of lignin. Lignin is a macromolecule which consists of phenolic units and is located in the cell walls of wood^23^. Odour-active phenyl derivatives identified in the samples are vanillin (*vanilla-like*), 3-phenylpropanoic acid (*vomit-like, fruity*), phenylacetic acid (*honey-like*) and p-cresol (*horse-like*). Furthermore, thymoquinone, which smells *pencil-like*, can be included in this group as the formation of quinones results from the oxidation of phenols. Thymoquinone as a naturally occurring, pencil-like smelling substance is a new find in pine wood. However, in our recent study^15^ on incense cedar wood, thymoquinone was found to have an even higher odour potency, possibly being one of its key odorants. In pine wood the attribute pencil-like was rated only with an intensity of 1.0 during sensory evaluation whereas in incense cedar wood it was the most dominant odour impression^15^. This reveals that even in those samples where thymoquinone was detected by olfactometry, it did not impact the overall smell of the pine samples as other compounds were present with much more sensory impact, thereby obviously covering the smell impression of this compound.

Two substances had an androstenone-like, perfume-like smell. Their chemical structures could not be unequivocally identified. Mass spectral matching, however, with the NIST library proposed the substances (5β)-androst-2-en-17-one and androst-2,16-diene. However, their identity could not be satisfactorily confirmed due to a lack of respective reference compounds. At first sight, the appearance of such substances in plant material might be surprising. Nevertheless, sterols are known constituents in the extractable fraction of wood^23^. Accordingly, the formation of compounds with a steroid structure is possible, meaning that the substances that were found might indeed be at least structurally related to androstenone.

Three substances remained unknown. They were characterised by *rubber-like*, *leather-like* and *mint-like* smell impressions respectively. A mass spectrum could not be obtained as no relevant peak was observable. Accordingly, their concentration in the samples is below the instrumental detection limit and they are, moreover, superimposed by other odourless substances, despite having low odour thresholds and being detectable by the nose. It is interesting to note that the leather-like smelling substance (RI = 2300) had an odour quality similar to 4-methylphenol and other phenolic compounds such as 4-ethylphenol (*horse stable-like, faecal, phenolic*; RI2168) and 2,3-dimethylphenol (*phenolic, ink-like, leather-like*; RI 2105); therefore the prospected substance is likely to have a phenolic core moiety. Nevertheless, the RI values did not match these odorous substances.

A number of substances identified in the present study have previously been reported as odorants in toasted wood from diverse wood species, namely acacia, chestnut, cherry, ash and oak. Such wood is commonly used for manufacturing barrels for wine ageing^13^. In another study, Diaz-Maroto *et al*.^14^ performed investigations on aroma-active compounds in different oak woods. Some of the identified odour-active substances also correlate with those compounds that we detected in *Pinus sylvestris* L. wood. A series of odorants stemming from fat degradation such as hexanal, (*E*)-non-2-enal and butanoic acid as well as phenolic derivatives such as p-cresol and vanillin have been reported as odorants in woods. However, as previous investigations on wood odorants were mainly based on hardwood used for barrel-making, this study provides the first targeted characterisation of odorous substances in pine wood. Pine wood contains a higher amount of resin. Thus, more terpenoic substances and higher amounts of terpenes are present in pine wood^24^. This substance class is already known as a source of volatiles in pine wood, contributing to its odour: For example, several terpenoids have been detected and identified as important odorants in incense cedar wood (e.g. thymoquinone, fenchol, α-bisabolol and γ-octalactone), which is also a softwood^15^. Others have generally been reported as volatiles in wood without further specification of their smell properties and their overall impact on the respective wood aroma (e.g. α-pinene, borneol)^9^.

Nevertheless, in the present study several odour-active compounds have been identified for the first time. A total of 11 substances were identified for the first time as odorants not only in pine wood but in wood in general: linalool (*citrusy*), (*E)*-dec-2-enal (*fatty*), borneol (*moldy*), pentanoic acid (*cheesy*), pentanal (*grassy*), dodecanoic acid (perfume-like, soapy), (*E,Z;Z*)-trideca-2,4,7-trienal (fruity, blood-like, metallic), γ-octalactone, δ-nonalactone (*coconut-like*), γ-decalactone and γ-dodecalactone (both *peach-like*). Hitherto, lactones such as these were only described as aroma compounds in relation to wine ageing in oak barrels and were only recovered from the aged wine and not from the wood itself, meaning there was no confirmation of their origin in the wood material^25^.

## Conclusion

The odorants identified in pine wood have great structural and sensorial diversity. Comparison of the gas chromatographic-olfactometric and sensory data showed there was close correlation between the respective sensory findings. Our study sheds light on the common odorants responsible for the characteristic smell of wood. This opens up opportunities for studying in more detail the pathways of wood odour formation, for optimizing processing strategies in order to positively influence and maintain wood smell quality, and for investigating artefact formation that leads to unpleasant or undesired smells in wood products. Moreover, these studies provide the basis for further research targeted at improving our understanding of the impact of wood smell (and its constituents) on the wellbeing of humans.

## Materials and Methods

### Chemicals

Dichloromethane (DCM) was purchased from VWR International GmbH (Ismaning, Germany) and freshly distilled prior to use in the wood odour extraction procedure. Anhydrous sodium sulphate was also obtained from VWR. Sodium hydrogen carbonate and hydrochloric acid were from Sigma-Aldrich (Steinheim, Germany). Reference substances were purchased from the following suppliers: 2,6,6-trimethylbicyclo[3.1.1]hept-2-ene (α-pinene) (97%), hexanal, octanal, 1-octene-3-one (50%), (*E)*-oct-2-enal (94%), acetic acid (>99%), (*Z)*-non-2-enal, (*E*)-non-2-enal (97%), (*E,Z)*-nona-2,6-dienal (95%), 3-methylbutanoic acid (99%), (*E,E)*-nona-2,4-dienal (85%), hexanoic acid (99.5%), 5-pentyloxolan-2-one (γ-nonalactone) (>98), 4-methylphenol (p-cresol) (99%), 6-butyloxan-2-one (δ-nonalactone) (98%), 5-hexyloxolan-2-one (γ-decalactone) (98%), dodecanoic acid (98%), phenylacetic acid (99%), nonanoic acid (97%), pentanal, 3,7-dimethylocta-1,6-dien-3-ol (linalool) (97%), heptanoic acid (99%), 4-hydroxy-3-methoxybenzaldehyde (vanillin) (99%), 3-hydroxy-4,5-dimethylfuran-2(5*H*)-one (sotolone) (97%), 2-isopropyl-5-methylbenzo-1,4-quinone (thymoquinone) (99%) and (2*S*)-6-methyl-2-[(1*S*)-4-methylcyclohex-3-en-1-yl]hept-5-en-2-ol (α-bisabolol) from Sigma Aldrich/Aldrich (Steinheim, Germany). Nonanal (95%), butanoic acid (>99.5%), pentanoic acid (99%), (*E,E*)-deca-2,4-dienal (85%), (*E)*-dec-2-enal (95%), (1*R*,2*R*,4*S*)-1,3,3-trimethyl-2-norbornanol (fenchol) (99%) and (1*S*,2*R*,4*S*)-1,7,7-trimethylbicyclo[2.2.1]heptan-2-ol (borneol) (99%) were purchased from Fluka (Steinheim, Germany), 5-butyl-4,5-dihydro-3*H*-furan-2-one (γ-octalactone) from EGA Chemie (Steinheim, Germany), 6-pentyloxan-2-one (δ-octalactone) (98%) from ABCR (Karlsruhe, Germany), 3-phenylpropanoic acid, dihydro-5-octyl-2(*3H*)-furanone (γ-dodecalactone) and 6-heptyltetrahydro-*2H*-pyran-2-one (δ-dodecalactone) from SAFC (Steinheim, Germany). *(E,Z,Z*)-Trideca-2,4,-trienal was synthesized according to Blank *et al*.^26^.

### Wood samples

The wood of three different trees of *Pinus sylvestris* L. was collected in the Reichswald Nürnberg, dried, and contemporarily planed into wood shavings. The received splints were about 20 cm long and 2 cm broad, while being less than 1 mm thick and could directly be used for further extraction and analysis.

### Human sensory analysis

In a first sensory analysis 30 untrained panellists (16 females, 14 males) were asked to rate pleasantness and intensity of the pine wood on a 10 cm visual analogue scale from 0 (no perception, very unpleasant) to 10 (very intense, very pleasant). The age of the panellists was from 22 to 43 years. A second sensory test was conducted with a trained panel from the Fraunhofer Institute for Process Engineering and Packaging IVV to describe the pine wood odour. The panel consisted of ten females and one male in the range of 23 to 53 years and was trained over at least 6 months to correctly identify and name odours during weekly training sessions with selected suprathreshold aroma solutions. The panellists were asked to name attributes for the perceived odour qualities of the wood samples, of which 12 attributes were chosen in consensus for latter intensity rating. In three following sensory sessions the odour quality of each wood sample was described using the given attributes, thereby rating their respective intensities on a 10 cm visual analogue scale from 0 (no perception) to 10 (strong perception).

### Solvent-assisted flavour evaporation (SAFE) of volatiles

2.5 g of the wood shavings of each of the three wood samples were extracted with 100 ml dichloromethane at room temperature for 30 min, filtered, and washed with another 10 ml of dichloromethane in each case, rendering three extracts in total. The resulting extracts were transferred to a solvent assisted flavour evaporation (SAFE) system^27^. The obtained distillate was dried over anhydrous sodium sulphate and concentrated at 50 °C to 100 µl using Vigreux-distillation and subsequent micro-distillation according to Bemelmans^28^. To ensure comprehensive recovery of the odorants, the third sample was additionally treated according to the following protocol: extraction with dichloromethane was conducted with 5 g of the wood shavings and stirring for 2 h in 100 ml dichloromethane. To improve resolution of all constituents during the subsequent GC-analysis, the extract was separated into a neutral-basic and an acid fraction using a technique from Buettner^29^. The extract was therefore shaken three times in a separation funnel with a total of 200 ml of a 0.5 mol NaHCO_3_-solution. The pH-value of the aqueous phase was adjusted to 2 by adding HCL dropwise. In the following, the acidic constituents were solvent-extracted using a total of 200 ml dichloromethane. The further treatment consisting of SAFE distillation and concentration by Vigreux- and micro-distillation was performed as described above. Following this procedure, two extracts of one sample were obtained. Since no additional odorants were found using this approach, the simple extraction without any further phase separation was proven to be sufficient for identification purposes. Since odorous substances are volatile and to avoid formation of artefacts, the temperature during sample workup was not raised higher than 55 °C (in the course of the distillation steps), and was commonly kept at room temperature. To further ensure that the applied extraction method is suitable for acquisition of a representative aroma extract, a drop of the undiluted final extract was brought on filter paper, and checked via sensory evaluation for its capability of eliciting the same odour impression as the original wood sample. The concentrated and diluted samples were stored at −80 °C and analysed within 4 weeks maximum, but commonly as freshly as possible.

### Gas chromatography-flame ionization detection/olfactometry (GC-O)

GC-O experiments were performed on different Trace GC Ultra (Thermo-Fisher Scientific GmbH, Dreieich, Germany) systems equipped with a DB-FFAP capillary (30 m × 0.32 mm, film thickness 0.25 µm, Agilent Technologies, Santa Clara, USA). Samples were applied using the cold-on-column technique. Therefore, 2 µl of the extracts were injected manually on a pre-column (deactivated fused silica capillary, 2–3 m length, 0.32 mm i.d.) at 40 °C. The pre-column was changed regularly to avoid accumulation of contaminants. With a Y-splitter attached to the end of the analytical capillary, the effluent was split in a 1:1 volume ratio and transferred to the flame ionization detector (FID) and the sniffing port, respectively, using two uncoated, deactivated fused silica capillaries (0.7 m × 0.32 mm).

The temperature programs used were as follows: The initial temperature of 40 °C was held for 2 min and then raised with a rate of 10 °C/min to a final temperature of 240 °C which was then held for 5 min. The helium carrier gas was at a constant flow of 2.2 ml/min.

### Aroma extract dilution analysis (AEDA)

The odorants in the obtained aroma extracts were analysed by means of aroma extract dilution analysis (AEDA)^16^. Therefore, the aroma concentrates were diluted stepwise 1 + 2 (v/v) with dichloromethane and resulted in a dilution series of 1:3^n^. The solutions were named after their corresponding flavour dilution factor (FD), calculated by 3^n^. For each odorant the FD factor was determined on a DB-FFAP column using GC-O. Thereby, the FD factor represents the last dilution step in which the odour of the corresponding substance was still detectable. The samples were sniffed by three different trained panellists, exhibiting no known illness at the time of examination to ensure that no important odorants were missed due to partial anosmia or lower sensitivities for particular substances. Additionally a blank extract was evaluated; thereby, it was found that the detected trace odorous contaminants did not have any relevant impact on the results of the AEDA experiments. The detected odorants representing the highest FD factors (FD ≥ 9) were then identified by means of gas chromatography-mass spectrometry/olfactometry (GC-MS/O) and two-dimensional gas chromatography- mass spectrometry/olfactometry (2D-GC-MS/O).

### High resolution gas chromatography–mass spectrometry/olfactometry (GC-MS/O)

The system used for identification of compounds was a Finnigan Trace GC Ultra (Thermo Electron Corporation/Thermo Scientific) coupled to a Thermo DSQ Single Quadrupol MS (Thermo Electron Corporation/Thermo Scientific), equipped with a Gerstel MPS 2 auto sampler. The software for mass spectral recording and data analysis was the Xcalibur Data System (Version1.4, Thermo Electron Corporation/Thermo Scientific). The analytical capillary used was a DB-FFAP (30 m × 0.25 mm, film thickness 0.25 µm, Agilent Technologies, Santa Clara, USA). An uncoated fused silica capillary was used as a pre-column (3 m × 0.53 mm) and changed regularly to avoid influences by accumulated impurities. The carrier gas was helium and the total flow was 3.3 mL/min. EI-mass spectra were generated in full scan mode (m/z range 40–400) using ionization energy of 70 eV. The starting temperature of 40 °C for the GC oven was held for 2 min, then raised at 8 °C/min to 240 °C and held for 5 min. Injection volumes were 2.0 µl.

### Heart-cut two-dimensional high resolution gas chromatography-mass spectrometry/olfactometry (2D-GC-MS/O)

The majority of the detected odorous compounds in wood were identified by means of heart-cut 2D-GC-MS/O. For this aim, the following set-up was used:

The analytical system consisted of two gas chromatographs (Varian CP-3800, Agilent Technologies, Santa Clara, USA) connected via a cryo trap system (CTS 1, Gerstel GmbH & CO KG, Mühlheim an der Ruhr, Germany). The first GC was equipped with a multi-column switching system MCS2 (Gerstel GmbH & CO KG, Mühlheim an der Ruhr, Germany). Analytical capillaries were DB-FFAP (first oven, 30 m × 0.32 mm film thickness 0.25 µm; Agilent Technologies, Santa Clara, USA) and DB-5 (second oven, 30 m × 0.32 mm, film thickness 0.25 µm; Agilent Technologies, Santa Clara, USA). An uncoated fused silica capillary (2–3.5 m × 0.53 mm) was used in the first oven as pre-column. Sample injection settings were the same as described for GC-MS/O analyses.

The elution range containing the compounds of interest (selected by the injection of reference compounds or detected odour impressions at the ODP) was transferred from the first GC column into the cryo trap (cooled with liquid nitrogen to −100 °C) using the MCS system. After thermodesorption of the trap (250 °C), the analytes were transferred into the second oven and separated on a capillary column of different polarity. At the end of the second capillary column, the eluent was split into an ODP (290 °C) and a mass spectrometer (Varian), enabling a simultaneous generation of mass spectra and the perception of the corresponding odour qualities of the respective odorants. All split capillaries were made of uncoated, deactivated fused silica material. Mass spectra were generated in the EI-full scan mode (m/z range 40–400, 70 eV).

### Identification criteria

All substances were identified based on their odour qualities and intensities, their retention indices (RI) on a FFAP column, and their mass spectra. The calculation of the retention indices was based on a series of homologous alkanes (C_6_-C_31_, 50 µg/ml in pentane)^30^. These calculated RI values were compared to the RI values of reference compounds or literature data. Reference substances were analysed at concentrations in the range from 5 to 50 µg/ml in the course of all GC-analyses, correlating to their respective intensities in the samples. In case that a comparison of mass spectra to those of reference substances was not possible, due to lack of authentic standards, they were compared to NIST Mass Spectral Library (Version 2.0, National Institute of Standards and Technology, USA).

### Ethics statement

The study was conducted in agreement with the Declaration of Helsinki. The study (registration number 180_16B) was approved by the Ethical Committee of the Medical Faculty, Friedrich-Alexander Universität Erlangen-Nürnberg. Informed consent was obtained from all subjects participating in the study.
